# Rapid and Sensitive Detection of Amino Groups in Chitosan Oligomers Using Aqueous Ninhydrin and McIlvaine Buffer

**DOI:** 10.3390/molecules31071101

**Published:** 2026-03-27

**Authors:** Oana Roxana Toader, Bianca-Vanesa Agachi, Andra Olariu, Corina Duda-Seiman, Gheorghita Menghiu, Vasile Ostafe

**Affiliations:** 1 Advanced Environmental Research Laboratories, West University of Timisoara, Oituz 4A, 300086 Timisoara, Romania; toaderor@gmail.com (O.R.T.); bianca.agachi@e-uvt.ro (B.-V.A.); vasile.ostafe@e-uvt.ro (V.O.); 2 Department of Biology, Faculty of Chemistry, Biology, Geography, West University of Timisoara, Pestalozzi 16, 300115 Timisoara, Romania; 3 Department of Scientific Research in Biology, Institute for Advanced Environmental Research, West University of Timisoara, Oituz 4C, 300086 Timisoara, Romania; 4 OncoGen Association, University of Medicine and Pharmacy “Victor Babes”, Aries 20, 300736 Timisoara, Romania; andraaolariu@gmail.com; 5 Department of Cellular and Molecular Biology, Faculty of Medicine, Titu Maiorescu University, Vacaresti 187, 031593 Bucharest, Romania; corina.seiman@prof.utm.ro

**Keywords:** chitooligomers, chitosan, ninhydrin, ultrasound-assisted solubilization, paper-based assay

## Abstract

Chitooligosaccharides (COS) are short-chain chitosan derivatives with a wide range of biomedical, agricultural, and environmental applications, including antimicrobial therapy, wound healing, and pollutant removal. Reliable quantification of COS is essential but currently relies on high-performance liquid chromatography, mass spectrometry, or capillary electrophoresis, which require costly equipment, complex sample preparation, and are unsuitable for routine or on-site applications. This study reports a rapid, solvent-free, colorimetric assay for COS based on the reaction of 5% aqueous ninhydrin with free amino groups in McIlvaine buffer. The assay was optimized using glucosamine as a model analyte, yielding maximal sensitivity at pH 7.0. The chromophore generated (Ruhemann’s purple) remained stable for over 120 min after reaction, allowing measurements to be taken without strict time constraints. Calibration was linear from 0.4 to 2.2 mM (R^2^ = 0.9926), with low limits of detection (0.006 mM) and quantification (0.018 mM). Increasing absorbance with COS polymerization degree (DP1–DP6) demonstrates specificity for free amino groups, while N-acetyl glucosamine showed a negligible response. Furthermore, the assay was successfully adapted for solid-phase detection on ninhydrin-pretreated filter paper and nitrocellulose, with enhanced sensitivity. This simple, efficient, and low-cost method provides an accessible alternative to instrumental techniques, supporting COS monitoring in laboratory workflows and enabling portable applications in biomedicine, agriculture, and environmental diagnostics.

## 1. Introduction

Chitooligosaccharides (COS) are short-chain derivatives of chitosan, obtained through partial hydrolysis of the β-(1-4)-linked D-glucosamine polymer. These compounds have gained significant attention due to their multifunctional biological properties [1]. In biomedical research, COS have been reported to exhibit antimicrobial activity against both Gram-positive and Gram-negative bacteria and to exert immunomodulatory effects that support wound healing and tissue regeneration [2,3]. In addition, COS and their derivatives have been investigated for anticancer-related properties, such as modulation of tumor cell proliferation and immune responses in both *in vitro* and *in vivo* models [4]. In pharmaceutical and technological contexts, COS are explored as functional ingredients owing to their favorable safety profile, biodegradability, and bioactivity. Several reviews have highlighted their potential applications in drug delivery systems, nutraceutical formulations, and functional food products, where reliable monitoring of COS composition and concentration is essential for formulation development and quality assurance [3,5]. In agriculture, COS are recognized as plant defense elicitors capable of enhancing resistance to biotic and abiotic stress. COS-induced activation of plant defense pathways and stress-related signaling has been proposed as a sustainable strategy for improving crop resilience, which requires controlled application and monitoring of COS-based treatments [6,7]. In food science and industrial applications, COS and their derivatives are studied as bioactive food additives and functional components. However, challenges related to stability, compositional consistency, and regulatory acceptance persist, underscoring the need for reliable analytical tools to monitor COS quality during processing and product development [3]. Beyond biomedical and agricultural uses, COS-based materials have also been explored for environmental and biotechnological applications, including pollutant binding and biopolymer-based remediation strategies. In these contexts, rapid screening methods are particularly valuable for assessing the presence and relative abundance of bioactive COS fractions during product development and process optimization [3,5]. Consequently, COS are increasingly employed in biomedical, pharmaceutical, agricultural, and environmental applications.

Accurate detection and quantification of chitooligosaccharides are essential for both fundamental research and practical applications. In industrial and applied settings, quality control of COS production is essential, as their biological and functional properties are strongly influenced by composition, degree of polymerization, and purity, which depend on extraction and processing conditions [8,9,10]. Although COS typically exert biological activity at nanomolar to micromolar concentrations in biological assay systems, they are commonly handled at micromolar to millimolar levels during production, formulation, and quality control workflows [4,6,7,9,11,12]. However, current analytical approaches, such as high-performance liquid chromatography (HPLC), mass spectrometry, and capillary electrophoresis, often require expensive equipment, long analysis time, and complex sample preparation, limiting their accessibility in resource-limited settings [11,12,13,14,15]. As a result, the development of a rapid, simple, and cost-effective analytical methods suitable for routine laboratory use and on-site detection remains a significant challenge.

Colorimetric assays, particularly those based on the ninhydrin reaction with free amino groups, offer a simple and cost-effective alternative for amino sugar detection. Ninhydrin is a well-established reagent for detecting primary amines in proteins, peptides, and free amino acids, with applications in agricultural and food quality control (determining amino acid content in dairy and meat products) and pharmaceutical analysis (quantifying amino-containing drugs and excipients) [16,17,18]. It is also widely employed in forensic science for latent fingerprint detection through its reaction with amino acids present in skin secretions [19,20]. However, only a few studies have applied ninhydrin to chitooligosaccharides, and those relied on organic solvents, such as ethanol, glacial acetic acid, or phosphoric acid, combined with laboratory-intensive protocols, which limit their practical applicability [21,22,23,24]. While ninhydrin is a well-established reagent for detecting primary amines through the formation of the purple Ruhemann’s chromophore, its adaptation for COS molecules with distinct structural and chemical characteristics compared to other amino-containing biomolecules has received limited attention. In particular, the potential of using aqueous ninhydrin solution under mild processing conditions for COS detection remains largely unexplored.

The present work addresses this gap by developing and validating a rapid aqueous ninhydrin assay for COS detection. The McIlvaine buffer was systematically evaluated to establish suitable reaction conditions, ensuring high sensitivity and reproducibility while avoiding the use of organic solvents. The method was further extended to detect COS directly on solid supports, such as filter paper and nitrocellulose membranes, enabling fast screening without the need for liquid-phase instrumentation. This approach combines the simplicity of colorimetric detection with enhanced reaction efficiency provided by ultrasound, offering a practical and scalable tool for COS analysis in both laboratory and on-site applications.

## 2. Results and Discussion

### 2.1. Optimization of pH and Product Stability

To establish a reliable and practical colorimetric method for chitooligosaccharides (COS) detection, the influence of key experimental parameters was systematically evaluated, including buffer pH, reaction product stability, calibration performance, and applicability across COS with different degrees of polymerization. The focus was on optimizing assay conditions to ensure both sensitivity and reproducibility while maintaining simplicity and avoiding the use of organic solvents or harsh processing steps. The results highlight the critical role of pH in modulating the ninhydrin–amino groups reaction, demonstrating the stability of Ruhemann’s purple chromophore under near-neutral conditions, and validating the assay through calibration curves, oligomer selectivity, and detection on solid supports.

**Figure 1:**
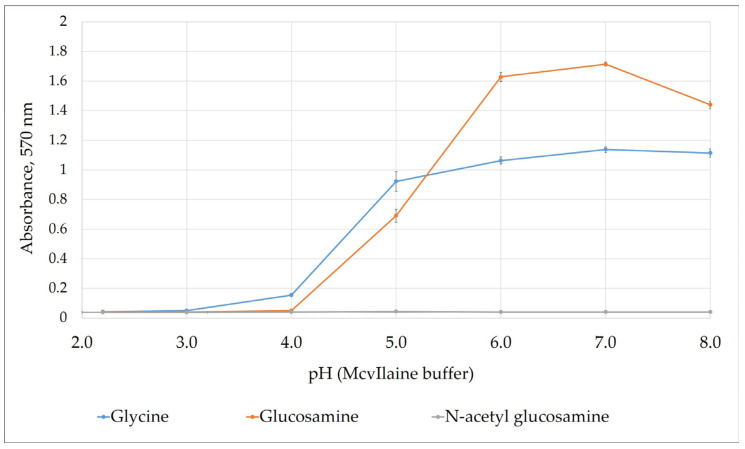
Effect of the McIlvaine buffer pH on the absorbance of glucosamine, glycine (positive control), and N-acetyl glucosamine (negative control) after reaction with 5% (*v*/*w*) aqueous ninhydrin. Data points represent mean values ± standard deviations from triplicate experiments (*n* = 3).

The influence of pH on the ninhydrin reaction was first investigated using glucosamine as the target sample, glycine as a positive control, and N-acetyl glucosamine as a negative control (Figure 1) using a McIlvaine buffer [25]. In this study, glycine was included as a positive control due to its free primary amino group and the well-established reactivity of amino acids with ninhydrin. Glucosamine (DP1) was selected as the calibration standard, as it contains a single free primary amino group and represents the basic structural unit of chitooligosaccharides. N-acetyl glucosamine was used as a negative control, since acetylation of the amino group prevents its reaction with ninhydrin, thereby enabling assessment of the assay specificity toward free primary amino groups. A clear increase was observed when the pH was raised from acidic to near-neutral values, with maximum color development reaching a pH of 6.0–7.0 for both glucosamine and glycine. In contrast, N-acetyl glucosamine produced negligible absorbance across the entire pH range, confirming the assay selectivity towards free amino groups. Interestingly, glucosamine displayed a sharper increase in absorbance between pH 6.0 and 7.0 compared to glycine, likely reflecting the influence of its amino acid sugar backbone on reaction kinetics. At pH values above 7.0, a slight decrease in absorbance was recorded for glucosamine, suggesting partial instability of the chromophore under alkaline conditions. These findings indicate that pH 7.0 provides an optimal balance for COS detection, ensuring high sensitivity and reproducibility while eliminating the need for strongly acidic or organic solvent-based systems commonly used in conventional ninhydrin assays.

**Figure 2:**
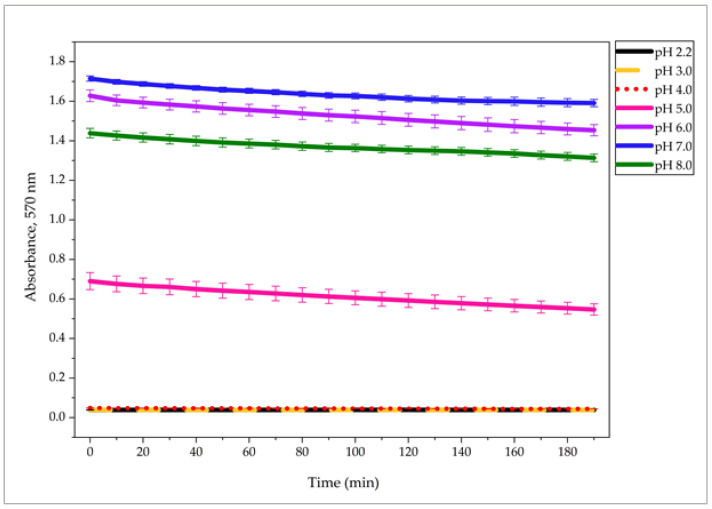
Stability of Ruhemann’s purple product formed in the ninhydrin–glucosamine reaction at different pH values, monitored at 570 nm for 190 min. Data points represent mean values ± standard deviations from triplicate experiments (*n* = 3). Corresponding reproducibility data are provided in Table S1 (Supplementary Materials).

The stability of Ruhemann’s purple product generated from glucosamine at different pH values (2.2, 3.0, 4.0, 5.0, 6.0, 7.0, and 8.0) was monitored for 190 min (Figure 2). In strongly acidic environments (pH 2.2–4.0), absorbance was negligible and unstable, confirming the unsuitability of these conditions for COS detection. In contrast, at pH 6.0–8.0, the reaction produced significantly higher absorbance values, followed by a slight and gradual decrease over time. No abrupt signal loss was observed at any of these pH values, and the reduction in absorbance was modest (approximately 5–10% over 190 min), suggesting satisfactory stability of the formed chromophore. Among the tested conditions, pH 7.0 yielded the highest initial absorbance and maintained a strong, consistent signal through the experiment, with only a minimal and nearly linear decline over time. This behavior indicates both efficient chromophore formation and good temporal stability. At pH 5.0, a similar gradual decrease was observed, although the overall stability and signal consistency were inferior compared to pH 7.0. Overall, the results demonstrate that near-neutral pH not only maximizes sensitivity (Figure 1) but also ensures time-based sensitivity of the reaction product, supporting pH 7.0 as the optimal conditions for assay performance.

Assessment of chromophore stability over time was performed to determine whether the assay requires immediate readout or allows flexible measurement timing. Stable color development supports the validity of the method for routine use and potential remote or delayed analysis.

### 2.2. Calibration Curve and Limit of Detection (LOD) for Glucosamine

**Figure 3:**
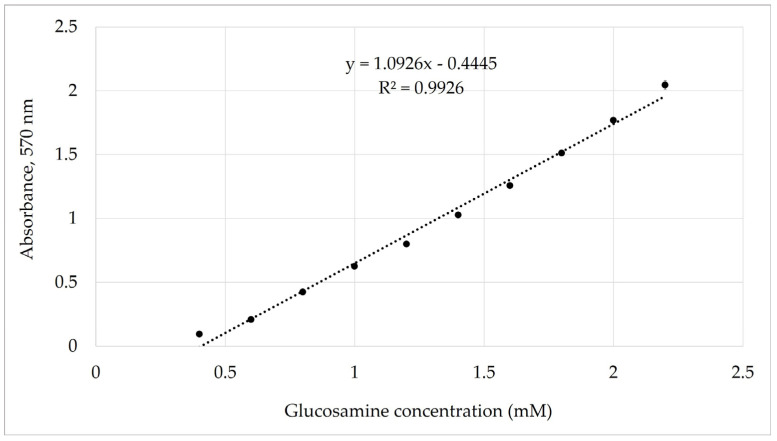
Calibration curve for glucosamine (0.4–2.2 mM) in the McIlvaine buffer (pH 7.0) measured at 570 nm. Linear regression was performed within the validated linear range, excluding the blank (0 mM) and the lowest concentration level (0.2 mM), which exhibited deviation from linearity near the detection limit. The regression equation was absorbance = 1.0926 × concentration − 0.4445 (R^2^ = 0.9926). The slight non-zero intercept is consistent with known deviations from ideal Beer–Lambert behavior in systems involving chemical equilibria and multiple absorbing species, as supported by the visible spectrum presented in Figure S1 (Supplementary Materials). LOD and LOQ were calculated according to ICH Q2 (R1). Data points represent mean values ± standard deviations from triplicate measurements (*n* = 3).

Under the optimized conditions, the calibration curve for glucosamine exhibited linear behavior in the concentration range of 0.4–2.2 mM. The regression analysis, performed excluding the zero-concentration point, yielded the equation Y = 1.0926 × X − 0.444 with R^2^ = 0.9926 (Figure 3). The calibration curves did not pass exactly through the origin. According to the literature, strict adherence to the Beer–Lambert law is observed only under ideal conditions; for example, in the presence of a single absorbing species and constant molar absorptivity [26]. In systems involving chemical equilibria or multiple absorbing species, deviations at very low concentrations are commonly observed. In the present case, spectral analysis (Figure S1, Supplementary Materials) confirmed the presence of two absorbing species (around 400 nm and 570 nm), which may contribute to minor deviations from ideal linearity at low concentration levels. The limit of detection (LOD) and limit of quantification (LOQ) were calculated according to ICH Q2 (R1) [27] using the standard deviation (σ) of the lowest concentration within the linear range (0.4 mM) and the slope of the calibration curve (S). Based on this approach, the LOD and LOQ were determined to be 0.006 mM and 0.018 mM, respectively, indicating the high sensitivity of the assay for glucosamine.

A similar response was observed when calibration was performed at 400 nm, indicating that the absorbance at both wavelengths is directly dependent on glucosamine concentration.

### 2.3. Response of the Aqueous Ninhydrin Assay to Chitooligosaccharides with Different Degrees of Polymerization

**Figure 4:**
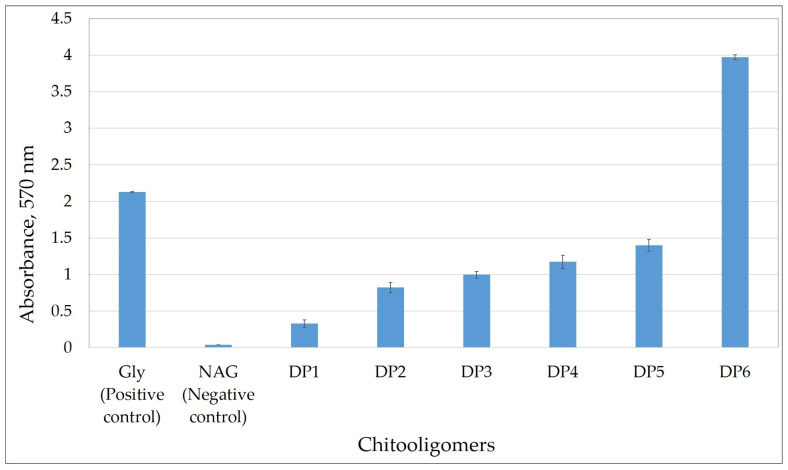
Microtiter plate colorimetric response of the aqueous ninhydrin assay to Gly (glycine, positive control), NAG (N-acetyl glucosamine, negative control), and chitooligosaccharides (DP1—glucosamine, DP2—chitobiose, DP3—chitotriose, DP4—chitotetraose, DP5—chitopentaose, DP6—chitohexaose) in the McIlvaine buffer (pH 7.0) at 570 nm. Data points represent mean values ± standard deviations from triplicate experiments (*n* = 3).

Chitooligosaccharides with degrees of polymerization (DP) from 1 to 6 were used in this study: glucosamine (DP1), chitobiose (DP2), chitotriose (DP3), chitotetraose (DP4), chitopentaose (DP5), and chitohexaose (DP6). The aqueous ninhydrin assay exhibited a clear and progressive increase in absorbance with the degree of polymerization of chitooligosaccharides (Figure 4). Negligible absorbance was observed for the negative control (N-acetyl glucosamine), confirming the specificity of the reaction for free amino groups. Glucosamine (DP1) produced a moderate response, while absorbance increased approximately linearly from chitobiose (DP2) to chitopentaose (DP5). Chitohexaose (DP6) generated the highest signal, with a more than threefold increase compared to glucosamine, highlighting the cumulative effect of multiple accessible amino groups on chromophore formation. These results demonstrate that the assay is sensitive to the degree of polymerization of COS, enabling not only their detection but also providing qualitative information on chain length under the optimized conditions.

In addition to chitooligosaccharides, the assay was evaluated on carboxymethyl chitosan, colloidal chitin, and water-soluble chitin. No detectable colorimetric response was obtained for these derivatives under the optimized conditions, indicating that the assay is selective for free primary amino groups and ineffective for highly substituted or insoluble chitin-based materials. This behavior reflects the need for accessible, non-acetylated amino functionalities for the ninhydrin reaction to occur. Although this limits the assay’s applicability to unmodified COS, it also highlights its utility for distinguishing bioactive oligomers from unreactive chitosan derivatives.

The calibration curve based on glucosamine was used as a general reference to evaluate the relative colorimetric response of COS with increasing degrees of polymerization (DP1–DP6).

### 2.4. Solid-Phase Detection of Chitooligosaccharides Using the Aqueous Ninhydrin Assay

**Figure 5:**
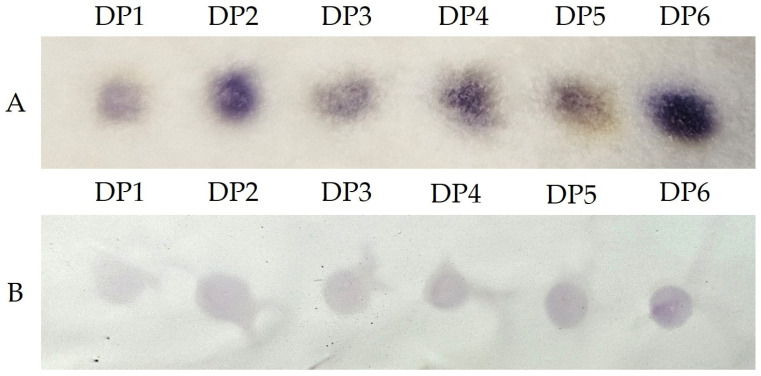
Detection of chitooligosaccharides (DP1–DP6) on solid supports using the aqueous ninhydrin assay. (**A**) Whatman filter paper pipetted with 5 µL of 1 mM chitooligosaccharides in the McIlvaine buffer (pH 7.0). (**B**) Nitrocellulose membrane pipetted with 1 µL of 1 mM chitooligosaccharides in the McIlvaine buffer (pH 7.0). Images shown are the most representative results selected from triplicate experiments, illustrating the qualitative colorimetric response of the assay on solid substrates.

The solid-phase assay shown in Figure 5 is intended as a qualitative screening approach and is inherently influenced by physical factors such as diffusion, adsorption, and substrate–analyte interactions. The aqueous ninhydrin assay enabled a clear visualization of chitooligosaccharides (DP1–DP6) on both filter paper and nitrocellulose membranes. Unlike the solution-based microtiter plate assay, where all COS are fully solubilized and the colorimetric response increases with the number of accessible amino groups, signal intensity on solid supports does not directly correlate with the degree of polymerization. Lower-DP COS (DP2) exhibit higher mobility and more homogenous diffusion within the filter paper matrix, which can result in visually stronger color development compared to intermediate DP species (DP3–DP5), while higher-DP species, such as DP6, generate sufficiently strong signals to remain detectable despite reduced diffusion. On nitrocellulose membranes, COS are readily detected; however, the visual response does not allow reliable discrimination between different degrees of polymerization under the tested conditions. Nevertheless, successful detection on two distinct solid substrates demonstrates the potential of this simple assay for rapid local screening of COS for biomedical, agricultural, and environmental purposes.

## 3. Materials and Methods

### 3.1. Reagents and Materials

Ninhydrin (4378.1), glucosamine hydrochloride (3769.1), chitobiose dihydrochloride (31TX.2), chitotriose trihydrochloride (336K.2), chitotetraose tetrahydrochloride (31X2.2), chitopentaose pentahydrochloride (31TY.2), chitohexaose hexahydrochloride (31X1.4), N-acetyl glucosamine (8993.2), chitin from shrimps (C7170), glycine (3790.2), hydrochloric acid (4625.1), sodium hydroxide (P031.2), citric acid (X863.2), disodium hydrogen phosphate (T877.1), and Whatman filter paper (1.0 mm thick) (HPL1.1) were purchased from Carl Roth, Karlsruhe, Germany. Amersham Protram Premium nitrocellulose membrane (0.45 µm pore size) (GE10600002) was bought from GE Healthcare Life Sciences, Little Chalfont, UK.

### 3.2. Preparation of 5% Aqueous Ninhydrin Solution

A 5% aqueous ninhydrin solution was prepared by dissolving 5 g of ninhydrin in 80 mL of distilled water under ultrasound in a water bath at 37 °C for about 30 min. The final volume was then adjusted to 100 mL with distilled water. The solution was stored at 4 °C and sonicated as needed prior to use.

### 3.3. Preparation of Solutions of Chitooligosaccharides and Their Derivatives

Chitooligosaccharides, including glucosamine hydrochloride, chitobiose dihydrochloride, chitotriose trihydrochloride, chitotetraose tetrahydrochloride, chitopentaose pentahydrochloride, and chitohexaose hexahydrochloride, were prepared as 0.1 M stock solutions in distilled water. Colloidal chitin and water-soluble chitin were prepared following the protocol published by Menghiu et al., 2019 [28]. Carboxymethyl chitosan and water-soluble chitin were each dissolved in distilled water to a final concentration of 0.1% (*w*/*v*), while colloidal chitin was prepared as a homogenous aqueous suspension at the same concentration.

### 3.4. Preparation of McIlvaine Buffer at Different pH Values

The McIlvaine buffer solutions were prepared following the original protocol [25]. Stock solutions of 0.1 M citric acid and 0.2 M disodium hydrogen phosphate were prepared separately. Buffers with pH values ranging from 2.2 to 8.0 were obtained by mixing specific volumes of phosphate and citric acid solutions, to reach a final volume of 20 mL: for pH 2.2–0.40 mL phosphate and 19.60 mL of citric acid, for pH 3.0–4.11 mL and 15.89 mL, for pH 4.0–7.71 mL and 12.29 mL, for pH 5.0–10.30 mL and 9.70 mL, for pH 6.0–2.63 mL and 7.37 mL, for pH 7.0–16.47 mL and 3.53 mL, and for pH 8.0–19.45 mL and 0.55 mL. Each mixture was thoroughly stirred until homogeneous, and the pH was verified with a calibrated pH meter from Mettler Toledo, Greifensee, Switzerland. The prepared buffers were stored at 4 °C and allowed to equilibrate to room temperature prior to use in the colorimetric assay.

### 3.5. Optimal Reaction pH in McIlvaine Buffer and Product Stability

Stock solutions (5 mM) of glycine (positive control), glucosamine (test compound), and N-acetyl glucosamine (negative control) were prepared in distilled water. For each assay, 20 µL of stock solution was dispensed into wells of a 96-well microtiter plate. The McIlvaine buffer (30 µL) adjusted to pH 2.2, 3.0, 4.0, 5.0, 6.0, 7.0, or 8.0 was then added, followed by 50 µL of 5% aqueous ninhydrin. The plate was incubated at 100 °C for 10 min in a HLC heating thermomixer MHR 11 from DITABIS AG, Reutlingen, Germany, and cooled for 10 min at room temperature. After that, the plate was mixed for 30 s, and the absorbance was measured at 570 nm using a BioTek Synergy H1 spectrophotometer (Agilent Technologies, Santa Clara, CA, USA). For stability assessment, absorbance of the reaction products was further monitored every 10 min for up to 190 min, under the same conditions.

### 3.6. Calibration Curve and Detection Limit for Glucosamine

A stock solution of 5 mM glucosamine was prepared in distilled water. Aliquots of 0, 2, 4, 6, 8, 10, 12, 14, 20, and 22 µL of the stock solution were combined with the McIlvaine buffer (pH 7.0) to a final volume of 50 µL in a microtiter plate. Subsequently, 50 µL of 5% (*w*/*v*) aqueous ninhydrin solution was added to each well. The reaction mixtures were incubated at 100 °C for 10 min and then cooled at room temperature. After incubation, absorbance was measured at 570 nm using a Biotek Synergy H1 spectrophotometer. Blank absorbance values were subtracted from all corresponding samples prior to data analysis to correct for the baseline contribution of the ninhydrin solution. The calibration curve was constructed by linear regression using glucosamine standards in the concentration range of 0.4 to 2.2 mM. The 0 mM (blank) and 0.2 mM concentration levels were excluded from regression analysis. The limit of detection (LOD) and the limit of quantification (LOQ) were calculated in accordance with the ICH Q2 (R1) guidelines [27] using the standard deviation of the response (σ) obtained from the lowest concentration within the validated linear range (0.4 mM) and the slope of the calibration curve (S) according to the equation LOD = 3.3 × (σ/S) and LOQ = 10 × (σ/S), where σ represents the standard deviation of replicate measurements at 0.4 mM and S is the slope of the regression line. The multipliers 3.3 and 10 are statistical factors recommended by ICH Q2 (R1) for estimating detection and quantification limits from calibration data. The resulting values represent the lowest glucosamine concentration that can be confidently detected and, respectively, quantified with acceptable precision and accuracy under the applied assay conditions.

### 3.7. Validation of Ninhydrin-Based Detection of Chitooligomers in McIlvaine Buffer

To further validate the previously developed ninhydrin-based assay for chitooligomers, 4 µL of each 10 mM chitooligosaccharide with degree of polymerization (DP) 1–6 (glucosamine, chitobiose, chitotriose, chitotetraose, chitopentaose, and chitohexaose) was mixed with 46 µL of the McIlvaine buffer at pH 7.0, to which 50 µL of 5% aqueous ninhydrin solution was added. The plate was then treated under the same conditions as in Section 3.6, including incubation and absorbance measurement.

### 3.8. Rapid Detection of Chitooligomers on Nitrocellulose and Filter Paper Using the Ninhydrin–McIlvaine Assay

Nitrocellulose strips and Whatman filter paper pieces (Sigma-Aldrich, St. Louis, MO, USA) were first immersed in 5% aqueous ninhydrin solution and allowed to dry slowly at room temperature. Then, 5 µL of each chitooligosaccharide solution (initially 10 mM, diluted 10-fold in McIlvaine buffer, pH 7.0) was pipetted onto pretreated filter paper, while 1 µL was applied to the nitrocellulose membrane. The strips were then gradually heated to 100 °C on a magnetic heating plate. The resulting colored spots, corresponding to the pipetted chitooligomers, were visually observed and photographed.

## 4. Conclusions

This study presents a rapid aqueous ninhydrin assay for the detection of chitooligosaccharides. Optimization of pH conditions revealed maximum sensitivity and stability of the chromogenic reaction at pH 7.0 in the McIlvaine buffer. The method exhibited excellent linearity for glucosamine (0.4–2.2 mM), with low detection (0.006 mM) and quantification (0.018 mM) limits, demonstrating high analytical sensitivity. Increasing responses with the degree of polymerization (DP1–DP6) of chitooligosaccharides confirmed the assay`s specificity for free amino groups, while successful qualitative application on filter paper and nitrocellulose validated its potential for simple, instrument-free, and on-site COS screening. These findings highlight the assay as a cost-effective, versatile tool for biomedical, agricultural, and environmental applications involving chitosan-derived products.

## Data Availability

The original contributions presented in this study are included in the article and Supplementary Materials. Further inquiries can be directed to the corresponding author.

## Supplementary Materials

The following supporting information can be downloaded at: https://www.mdpi.com/article/10.3390/molecules31071101/s1, Figure S1: Visible absorption spectra (330–700 nm) of glucosamine reacted with ninhydrin in McIlvaine buffer and the corresponding negative control (McIlvaine buffer); Table S1: Mean absorbance values and standard deviations of Ruhemann’s purple formed in the reaction between glucosamine and ninhydrin, monitored over time (0–190 min, 10 min intervals) at different pH values (A 570 nm, mean ± SD, *n* = 3).
