# Supplementary material for: Rapid and Sensitive Detection of Amino Groups in Chitosan Oligomers Using Aqueous Ninhydrin and McIlvaine Buffer
Source: Molecules. 2026 Mar 27;31(7):1101. doi: 10.3390/molecules31071101 (PMC13074245; doi:10.3390/molecules31071101)
Supplement: Supplementary file 1 [file molecules-31-01101-s001.zip › molecules-3874521-supplementary.pdf]

Table S1. Mean absorbance values and standard deviations of Ruhemann`s purple formed in the reaction between glucosamine and ninhydrin, monitored over time (0-190 min, 10 min intervals) at different pH values (A 570 nm, mean ± SD, *n*=3).

**Time (min)**	**pH 2.2**	**pH 3.0**	**pH 4.0**	**pH 5.0**	**pH 6.0**	**pH 7.0**	**pH 8.0**
**0**	0.0387 ± 0.0006	0.039 ± 0.0000	0.049 ± 0.0000	0.690 ± 0.043	1.628 ± 0.029	1.715 ± 0.013	1.439 ± 0.024
**10**	0.039 ± 0.0000	0.039 ± 0.0000	0.048 ± 0.0000	0.676 ± 0.040	1.605 ± 0.027	1.698 ± 0.012	1.427 ± 0.023
**20**	0.039 ± 0.0000	0.039 ± 0.0000	0.048 ± 0.0000	0.667 ± 0.039	1.593 ± 0.027	1.688 ± 0.012	1.417 ± 0.024
**30**	0.039 ± 0.0000	0.039 ± 0.0000	0.0477 ± 0.0006	0.661 ± 0.040	1.583 ± 0.027	1.677 ± 0.012	1.408 ± 0.024
**40**	0.039 ± 0.0000	0.039 ± 0.0000	0.0473 ± 0.0006	0.650 ± 0.039	1.574 ± 0.029	1.668 ± 0.012	1.399 ± 0.023
**50**	0.039 ± 0.0000	0.039 ± 0.0000	0.047 ± 0.0000	0.642 ± 0.038	1.564 ± 0.029	1.658 ± 0.013	1.391 ± 0.023
**60**	0.039 ± 0.0000	0.039 ± 0.0000	0.047 ± 0.0000	0.635 ± 0.038	1.556 ± 0.029	1.653 ± 0.013	1.386 ± 0.023
**70**	0.039 ± 0.0000	0.039 ± 0.0000	0.0463 ± 0.0006	0.628 ± 0.037	1.548 ± 0.029	1.646 ± 0.013	1.380 ± 0.022
**80**	0.039 ± 0.0000	0.039 ± 0.0000	0.046 ± 0.0000	0.620 ± 0.036	1.538 ± 0.030	1.637 ± 0.014	1.373 ± 0.021
**90**	0.039 ± 0.0000	0.039 ± 0.0000	0.046 ± 0.0000	0.613 ± 0.036	1.530 ± 0.030	1.630 ± 0.014	1.366 ± 0.020
**100**	0.0393 ± 0.0006	0.039 ± 0.0000	0.0457 ± 0.0006	0.606 ± 0.035	1.523 ± 0.032	1.627 ± 0.015	1.364 ± 0.019
**110**	0.039 ± 0.0000	0.039 ± 0.0000	0.0453 ± 0.0006	0.599 ± 0.035	1.515 ± 0.032	1.621 ± 0.016	1.358 ± 0.019
**120**	0.0397 ± 0.0012	0.039 ± 0.0000	0.045 ± 0.0000	0.592 ± 0.035	1.506 ± 0.032	1.613 ± 0.017	1.354 ± 0.020
**130**	0.039 ± 0.0000	0.039 ± 0.0000	0.045 ± 0.0000	0.586 ± 0.034	1.498 ± 0.033	1.608 ± 0.017	1.350 ± 0.019
**140**	0.039 ± 0.0000	0.039 ± 0.0000	0.045 ± 0.0000	0.579 ± 0.033	1.490 ± 0.034	1.603 ± 0.018	1.347 ± 0.020
**150**	0.039 ± 0.0000	0.039 ± 0.0000	0.0443 ± 0.0006	0.572 ± 0.032	1.482 ± 0.034	1.601 ± 0.019	1.342 ± 0.020
**160**	0.039 ± 0.0000	0.039 ± 0.0000	0.044 ± 0.0000	0.566 ± 0.032	1.474 ± 0.033	1.600 ± 0.021	1.336 ± 0.020
**170**	0.039 ± 0.0000	0.039 ± 0.0000	0.044 ± 0.0000	0.559 ± 0.031	1.467 ± 0.031	1.596 ± 0.021	1.328 ± 0.020
**180**	0.039 ± 0.0000	0.039 ± 0.0000	0.044 ± 0.0000	0.553 ± 0.030	1.460 ± 0.030	1.593 ± 0.020	1.321 ± 0.020
**190**	0.039 ± 0.0000	0.039 ± 0.0000	0.044 ± 0.0000	0.547 ± 0.029	1.454 ± 0.029	1.591 ± 0.018	1.314 ± 0.020

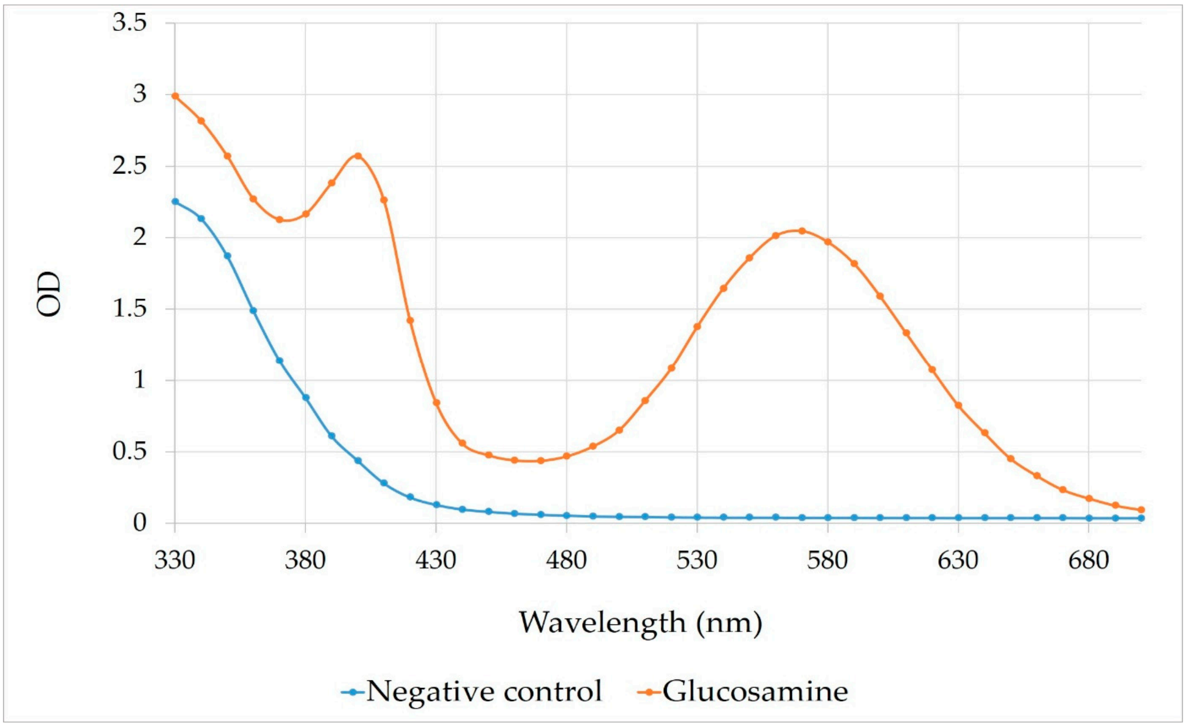

Figure S1. Visible absorption spectra (330-700 nm) of glucosamine reacted with ninhydrin in McIlvaine buffer and the corresponding negative control (McIlvaine buffer). The spectrum of the reaction mixture displayed two absorption maxima at approximately 400 nm and 570 nm. The bands are characteristic of Ruhemann`s purple, while the additional band at ~ 400 nm indicates the presence of other absorbing species within the reaction system. The negligible absorbance of the negative control confirms the selectivity of the chromogenic reaction.
